# Reconsidering the LARS score: a cross-sectional descriptive study exploring complementary screening approaches for low anterior resection syndrome

**DOI:** 10.1007/s00384-026-05115-9

**Published:** 2026-02-28

**Authors:** Yolanda Ribas, Ladislao Cayetano, Nuria Ortega-Torrecilla, Eloy Espín-Basany, Josep Bargalló, Clara Romero, Franco Marinello

**Affiliations:** 1 Coloproctology Unit, Consorci Sanitari de Terrassa, Terrassa, Barcelona, Spain; 2 Autonomous University of Barcelona, Barcelona, Spain; 3 Department of General and Digestive Surgery, Coloproctology Unit, Hospital Universitari Vall d’Hebrón, Barcelona, Spain; 4 Department of Research and Innovation, Consorci Sanitari de Terrassa, Terrassa, Barcelona, Spain

**Keywords:** Low anterior resection syndrome, LARS score, International Consensus Definition, Bowel dysfunction, Rectal cancer surgery

## Abstract

**Purpose:**

The LARS score is a practical tool to screen for bowel dysfunction after rectal cancer surgery. However, clinical experience suggests that it may overlook relevant symptoms and/or overestimate impact in some patients. This study aimed to explore whether the International Consensus Definition of LARS complements the LARS score in identifying patients with bowel dysfunction.

**Methods:**

We conducted a cross-sectional study including patients treated for rectal cancer across two hospitals between January 2021 and December 2024. Demographic and clinical data were collected retrospectively. Functional outcomes were assessed during outpatient follow-up using both the LARS score and the International Consensus Definition criteria.

**Results:**

Sixty-two patients were included. According to the LARS score, 39 (62.9%) had “no LARS”, 10 (16.1%) “minor LARS” and 13 (21%) “major LARS”. Using the International Consensus Definition, 24 (38.7%) met the criteria for LARS. Nine patients (14.5%) were classified differently by the two tools. Five patients classified as “no LARS” by the LARS score met the International Consensus Definition due to unpredictable bowel function and emptying difficulties with a reported impact on daily life. In contrast, four patients with “minor or major LARS” did not meet the International Consensus Definition criteria because no consequences were reported.

**Conclusion:**

In this exploratory cross-sectional cohort, the International Consensus Definition did not identify substantially more patients than the LARS score but provided complementary information by linking symptoms to their perceived consequences. Combining both tools may offer a more comprehensive appraisal of LARS until newer multidimensional instruments become available.

## Introduction

Low anterior resection syndrome (LARS) is a common and often disabling bowel dysfunction that may occur after anterior resection for rectal cancer. It is characterised by variable and unpredictable symptoms, including increased stool frequency, urgency, incontinence, and fragmented evacuation [1, 2]. LARS can significantly affect daily activities, quality of life, and mental wellbeing and may also impose additional economic burdens depending on the healthcare system [3–5].

The LARS score [6] is a simple questionnaire widely used as a screening tool for this condition. However, both clinical experience and previous studies [7, 8] have highlighted important limitations. It may fail to capture relevant symptoms—particularly severe evacuatory dysfunction—and it does not fully reflect the patient’s subjective perception of symptom burden. In one clinical study, 23.8% of patients with significant evacuatory problems were classified as “no LARS”, while 24% of those categorised as “major LARS” reported no perceived impact during a detailed clinical interview. Such discrepancies may lead to both underdiagnosis and overestimation in clinical practice.

The International Consensus Definition of LARS [9] represents a significant advance, offering a more comprehensive and patient-centred approach. Created through an international initiative involving patients and healthcare professionals, it integrates both symptoms and their consequences, ensuring that all key aspects of the patient experience are captured. Moreover, by separating symptoms from consequences, this definition allows for more precise detection of changes over time or in response to therapy, potentially improving evaluation strategies and personalised care.

To date, few studies have applied the International Consensus Definition alongside the LARS score in clinical cohorts. Nguyen et al. [10] incorporated both instruments in a remote needs assessment of patients after anterior resection, highlighting the importance of symptom-related consequences in identifying clinically relevant bowel dysfunction. Atsumi et al. [11] applied a study-specific questionnaire based on the International Consensus Definition in a multicentre cohort and reported that a subset of patients classified as “no LARS” by the LARS score nonetheless met definition-based criteria for bowel dysfunction.

However, the specific role of the International Consensus Definition as a complementary tool to the LARS score in clinical assessment remains unclear.

The primary aim of this study was to explore whether the International Consensus Definition complements the LARS score in identifying patients with bowel dysfunction.

## Methods

We conducted a cross-sectional study at two hospitals: Consorci Sanitari de Terrassa (Terrassa, Spain) and Hospital Universitari Vall d’Hebron (Barcelona, Spain). Patients who underwent surgery between January 2021 and December 2024 were identified from the Coloproctology Unit registries at both centres and assessed during a single outpatient visit. Demographic and clinical data were collected retrospectively from medical records after obtaining written informed consent, while functional outcomes (LARS score and International Consensus Definition) were evaluated at the time of the outpatient visit.

Inclusion criteria were total (TME) or partial mesorectal excision (PME) with colorectal or coloanal anastomosis, with ileostomy reversal at least 3 months before inclusion. Exclusion criteria were abdominoperineal resection, disseminated disease, non-reversed ileostomy, active chemotherapy, refusal to participate, or cognitive/language barriers preventing questionnaire completion.

Bowel function was assessed using the validated Spanish version of the LARS score [6, 12] and a Spanish translation of the International Consensus Definition [9], which was translated by the research team and reviewed by participating clinicians; no formal cross-cultural validation procedures were performed. The LARS score is a 5-item questionnaire that classifies patients as no LARS (0–20 points), minor LARS (21–29 points) or major LARS (30–42 points). The total score, proportion of patients in each category and individual symptoms were recorded. The LARS score also includes a dedicated item at the end of the questionnaire, with four response options, in which patients rate the perceived impact of bowel function on their quality of life; no separate validated quality-of-life questionnaire was administered. The International Consensus Definition incorporates both symptoms and their consequences, offering a broader, patient-centred perspective. The number of patients who met the definition “having undergone an anterior resection of the rectum and presenting at least one of the specified symptoms resulting in at least one of the specified consequences” was also documented, along with the frequency of individual symptoms and consequences.

Data management and statistical analysis were performed using IBM SPSS Statistics, v30.0 (IBM Corp., Armonk, NY, USA). Categorical variables are reported as counts and percentages (*n*, %). Age is reported as mean ± standard deviation (SD) and range (minimum-maximum).

The study was approved by the Ethics Committees of both participating institutions (approval numbers Consorci Sanitari de Terrassa 02–25–102–011; Hospital Universitari Vall d’Hebrón PR(AG)068/2025).

## Results

### Patient characteristics

A total of 62 patients were included, 34 from Consorci Sanitari de Terrassa (54.8%) and 28 from Hospital Universitari Vall d’Hebron (45.2%). Baseline characteristics and operative details are summarised in Table 1**.** Most procedures were performed laparoscopically or robotically, more than 50% had a total mesorectal excision (TME), most anastomoses were colorectal and more than half of patients had a temporary diverting ileostomy, of whom 60% underwent afferent loop stimulation before reversal. Five patients developed anastomotic dehiscence, all of whom were managed conservatively.

**Table 1:** Baseline characteristics and clinical data

Sex	Men 64.5%/women 35.5%
Age	70 ± 10 years (46–88)
Neoadjuvant treatment	35 (56.5%)
Surgical approach	Laparoscopic (29 (46.8%))
	Robot (28 (45.2%))
	taTME (5 (8.1%))
TME/PME	TME (33 (53.2%))
	PME (29 (46.8%))
Anastomosis	Colorectal (59 (95.2%))
	Coloanal (3 (4.8%))
Ileostomy	35 (56.5%)
Afferent loop stimulation	21/35 (60%)
Anastomotic dehiscence	5 (8.1%)
	Conservative management 3/5
	Endoscopic management 2/5
Adjuvant QT	27 (43.5%)
Previous treatments for LARS	25 (40.3%)
Diet changes	24 (38.7%)
Fibre	18 (29%)
Enemas	5 (8.1%)
Loperamide	7 (11.3%)

The mean follow-up since bowel reconstruction was 23 ± 12.5 months (range 3–49). Almost 40% of patients had made diet changes, and 25 (40%) had received some form of treatment for bowel dysfunction, mainly dietary fibre, enemas or medication (loperamide), but none had used transanal irrigation or sacral neuromodulation (Table 1).

### Bowel dysfunction according to the LARS score

**Fig. 1:**
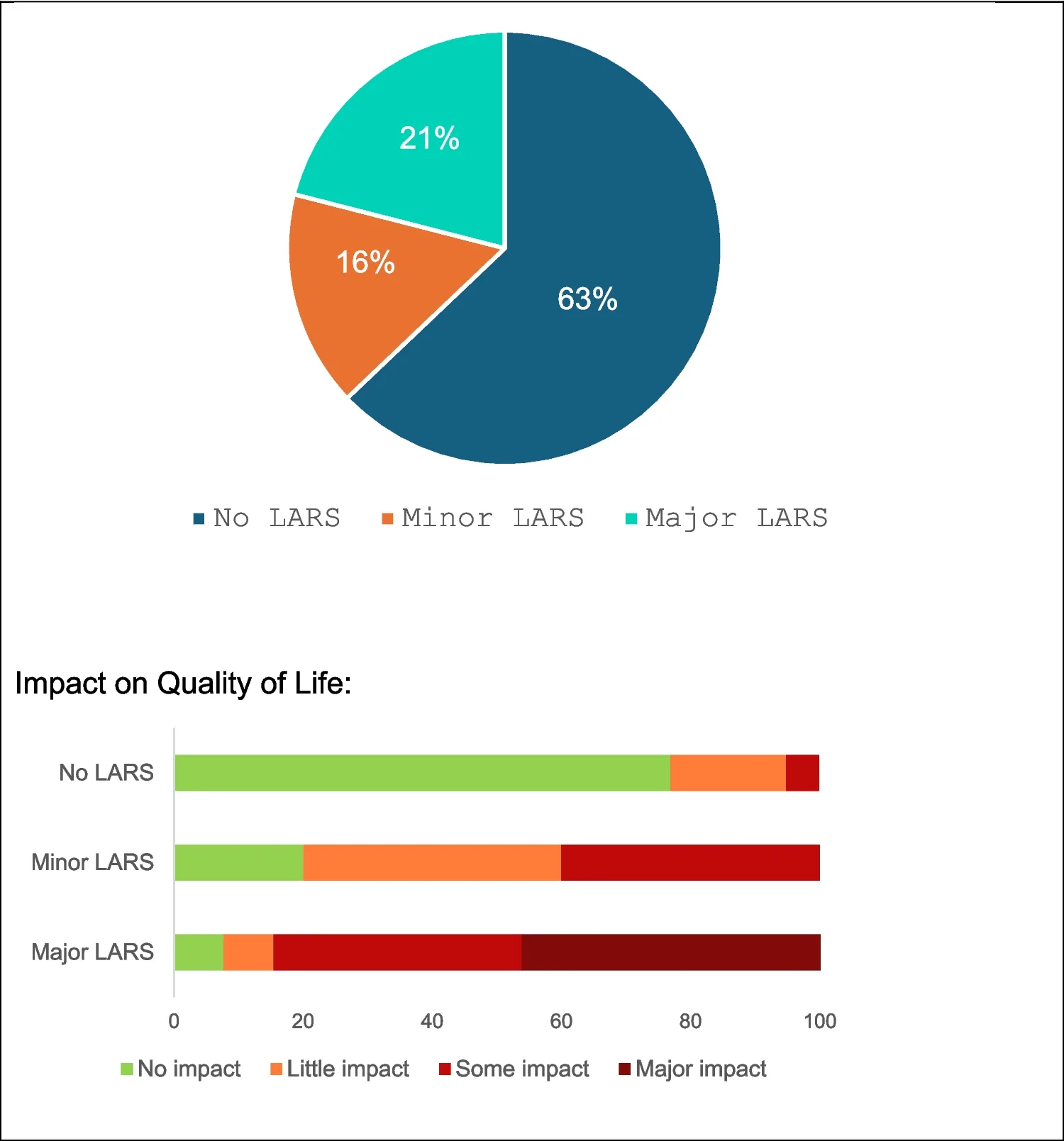
LARS categories according to the LARS score

**Fig. 2:**
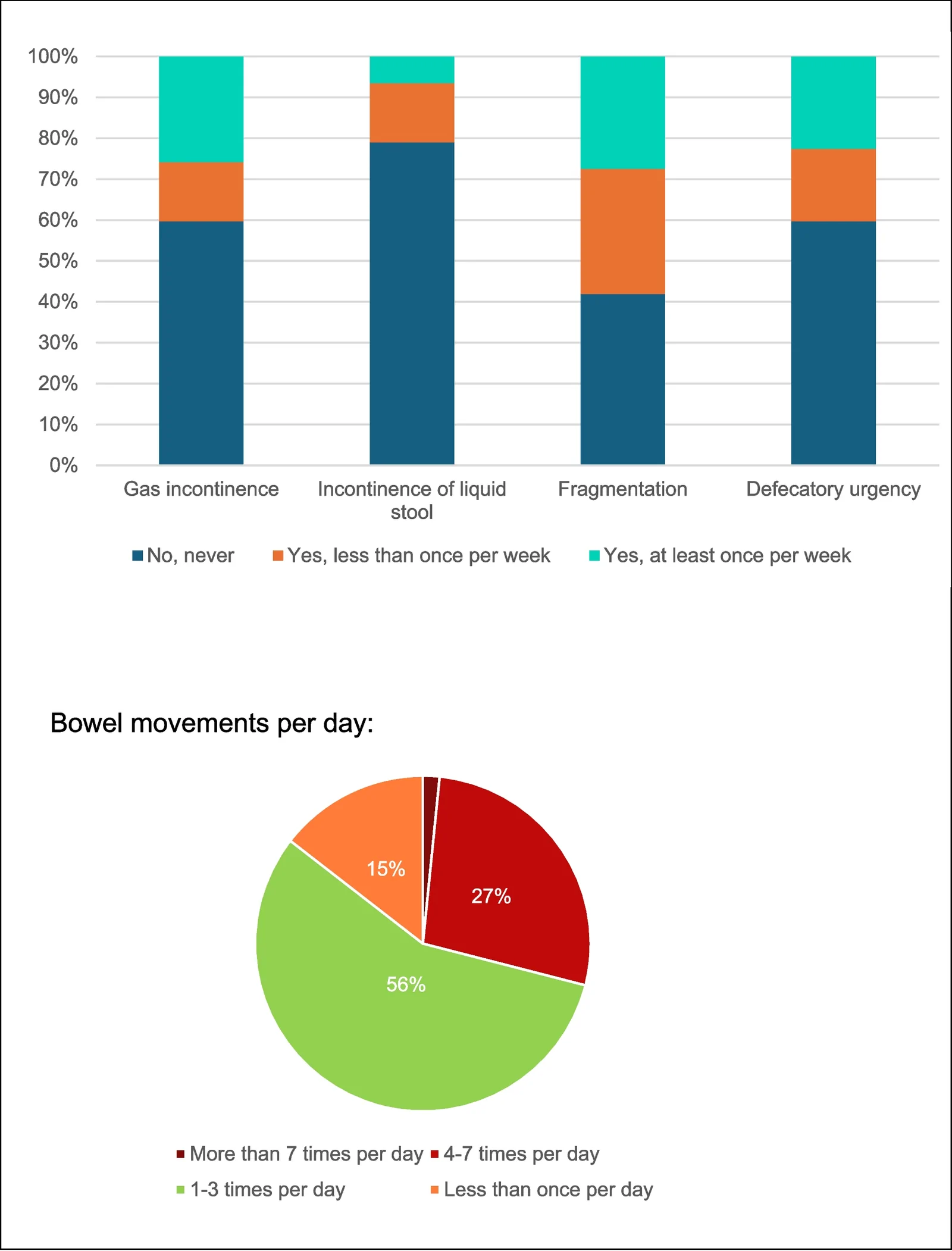
LARS symptoms according to the LARS score

The mean LARS score was 15.5 ± 13.3 (range 0–41). According to the classification, 39 (62.9%) patients were categorised as “no LARS”, 10 (16.1%) as “minor LARS”, and 13 (21%) as “major LARS” (Fig. 1). Figure 1 shows the impact on QoL rated by patients. Prevalence of LARS symptoms is shown in Fig. 2.

### Identification of bowel dysfunction according to the International Consensus Definition

Based on the International Consensus Definition, 24 patients (38.7%) met the criteria for LARS. The mean number of reported symptoms was 2.35 ± 2.25 (range 0–7), and the mean number of consequences was 1.56 ± 2.26 (range 0–8). The most prevalent symptoms were emptying difficulties and variable unpredictable bowel function. The most prevalent consequences were impact on strategies and compromises, dissatisfaction with bowels, and toilet dependence (Table 2).

**Table 2:** International Consensus Definition: symptoms and consequences

Low anterior resection syndrome			
Symptoms		Consequences	
Emptying difficulties	53.2%	Strategies and compromises	30.6%
Variable, unpredictable bowel function	43.5%	Dissatisfaction with bowels	27.4%
Altered stool consistency	32.3%	Toilet dependence	22.6%
Increased stool frequency	30.6%	Impact on social and daily activities	21%
Soiling	30.6%	Preoccupation with bowel function	19.4%
Urgency	27.4%	Impact on mental and emotional wellbeing	11.3%
Incontinence	12.9%	Impact on relationships and intimacy	11.3%
Repeated painful stools	8.1%	Impact on roles, commitments and responsibilities	11.3%

### Assessment of bowel dysfunction using both instruments

Our study evaluated the clinical applicability of the International Consensus Definition and its ability to complement the LARS score. Out of 62 patients included, 9 (14.5%) showed discordant results between the two tools (Table 3).

**Table 3:**
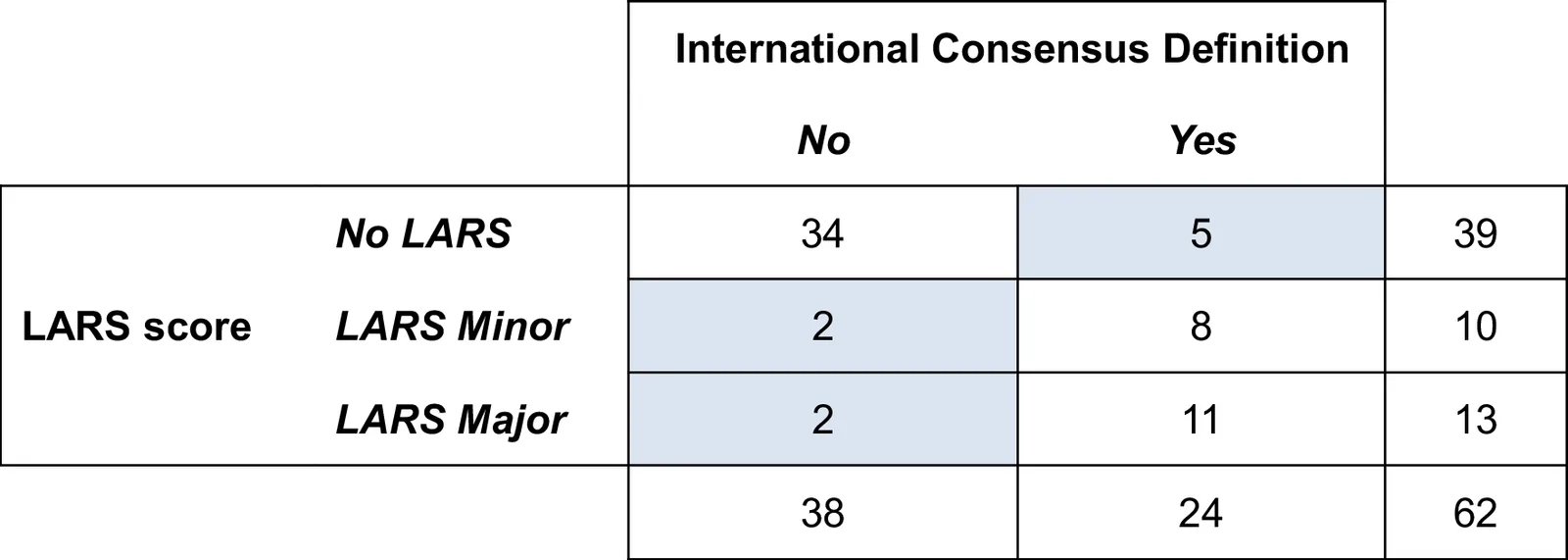
Assessment of bowel dysfunction using both instruments

Five patients were classified as “no LARS” according to the LARS score but met the International Consensus Definition criteria. All five reported emptying difficulties, and four also described unpredictable and variable bowel function.

Conversely, four patients classified as “minor” or “major LARS” according to the LARS score did not meet International Consensus Definition criteria, as they reported some symptoms but no consequences. In all four cases, patients had indicated “no impact” on quality of life in their LARS questionnaire.

## Discussion

This study explored whether the International Consensus Definition of LARS complements the LARS score, the current screening tool for bowel dysfunction after anterior resection, in identifying clinically relevant dysfunction. In a cohort of 62 patients, the two tools were largely concordant overall, with nine (14.5%) showing discordant classifications between the two tools. The International Consensus Definition identified five patients with LARS who were not detected by the LARS score, all of whom reported emptying difficulties, and most also described unpredictable and variable bowel function associated with reported consequences. In contrast, the LARS score classified four patients as having LARS even though no consequences were reported when assessed using the International Consensus Definition criteria. This is consistent with the findings of Nguyen et al. [10] who, in a needs assessment using mailed questionnaires, also reported substantial agreement between the two tools, with discordance largely driven by whether patients reported consequences affecting daily life. Similarly, Atsumi et al. [11] reported that a proportion of patients categorised as “no LARS” by the LARS score met criteria for bowel dysfunction using the International Consensus Definition, with variable or unpredictable bowel function being the most commonly reported symptom.

The LARS score is simple and pragmatic, but several limitations have become evident in routine use [7, 8]. In clinical practice, it is often used as the sole indicator of bowel dysfunction after rectal resection; however, this approach may fail to fully reflect the impact of symptoms on daily life. This limitation is particularly apparent in patients with highly variable bowel patterns—for example, several days without defaecation followed by a day with multiple, fragmented stools of varying consistency—who often find it difficult to provide a meaningful “average” frequency. As a result, episodic or fluctuating symptoms may be inadequately captured. Unpredictability may substantially disrupt daily life, even in otherwise active individuals.

Another important limitation is that the LARS score does not account for the consequences of symptoms. This may contribute to potential misclassification of clinical relevance: over-identifying patients with minimal impact and under-identifying those with disruptive symptoms, such as patients with evacuatory dysfunction [7, 13]. In addition, some patients may report little perceived impact on quality of life because they have developed effective coping strategies. For example, retired individuals or those with flexible routines may adapt their daily activities—such as leaving home only after ensuring they have fully emptied their bowels—which can mask the disruptive nature of their symptoms.

The International Consensus Definition provides a binary classification derived from a broader set of symptoms and consequences. In this series, it identified patients in whom unpredictable bowel habit and evacuatory difficulty had a tangible impact on daily activities, despite a “no LARS” classification on the LARS score. Conversely, patients classified as having “minor or major LARS” according to the LARS score did not meet the International Consensus Definition criteria in the absence of reported consequences.

While both tools aim to characterise bowel dysfunction after anterior resection, they represent conceptually different approaches: the LARS score grades symptom severity, whereas the International Consensus Definition focuses on clinically relevant consequences. Notably, despite its conceptual strengths, the International Consensus Definition has rarely been implemented in routine practice or research.

Moreover, the LARS score is based on an undefined recall period, which may lead to underestimation or omission of episodic or fluctuating symptoms. A previous study [14] comparing questionnaires with stool diaries has shown only weak correlations, particularly in patients with severe evacuatory dysfunction, suggesting that additional tools such as stool diaries may provide clinically relevant information with reduced recall bias. A multimodal approach combining questionnaires with stool diaries may therefore improve the functional assessment of patients with LARS and inform clinical decision-making, including the consideration of medical treatment.

This study has limitations inherent to its cross-sectional design and the relatively small sample size. Moreover, nearly half of the cohort underwent partial mesorectal excision (PME), which is generally associated with better functional outcomes than TME. This surgical heterogeneity may have contributed to the relatively low prevalence of “major LARS” observed, which may have attenuated discordance between instruments. We did not perform stratified analyses by extent of mesorectal excision (PME vs. TME); therefore, the direction and magnitude of this potential effect remain speculative.

In addition, the wide follow-up range (3–49 months) introduces temporal heterogeneity, as functional outcomes evolve over time and patients may develop coping strategies that modify symptom perception and reporting. This variability may have influenced response dispersion and the observed agreement between instruments. As no formally validated Spanish version of the International Consensus Definition was available, the criteria were translated into Spanish by the research team and reviewed by clinicians from the participating centres. However, no back-translation, pilot testing or formal cross-cultural validation procedures were performed, which represents a methodological limitation. Finally, participation bias cannot be excluded, as individuals experiencing fewer symptoms or consequences may have been less inclined to participate. Nevertheless, this design provides a pragmatic snapshot of how both instruments perform in routine clinical follow-up.

Overall, the findings of this exploratory cross-sectional study suggest that reliance on the LARS score alone may fail to identify a small subset of patients with clinically relevant bowel dysfunction, particularly those with evacuatory difficulties or unpredictable bowel patterns. Although the International Consensus Definition did not substantially increase case identification in this cohort, it helped to highlight patients in whom symptoms had meaningful consequences in daily life. In this sense, the two approaches may be considered complementary, as they capture different aspects of bowel dysfunction after anterior resection.

Given the significant impact of LARS on daily functioning and quality of life, accurate identification of affected patients is essential to enable appropriate clinical assessment and support. Until newer multidimensional instruments, such as the LARS iCAT score, become available and validated for routine use, careful clinical evaluation remains key to ensure that patients with relevant symptoms are not overlooked.

## Data Availability

The data supporting the findings of this study are not publicly available due to ethical and privacy restrictions, as they contain coded patient data. Data may be available from the corresponding author upon reasonable request and subject to institutional and ethical approval.
